# Stress-Induced Cholesterol Metabolic Dysregulation and Differentiation Trajectory Shift in Oligodendrocytes Synergistically Drive Demyelination

**DOI:** 10.3390/ijms26083517

**Published:** 2025-04-09

**Authors:** Weihao Zhu, Rui Shi, Yingmin Li, Guowei Zhang, Xiaowei Feng, Jingze Cong, Mengting He, Yuchuan An, Rufei Ma, Weibo Shi, Bin Cong

**Affiliations:** Department of Forensic Medicine, Hebei Key Laboratory of Forensic Medicine, Collaborative Innovation Center of Forensic Medical Molecular Identification, Hebei Medical University, Shijiazhuang 050017, China; 22031100049@stu.hebmu.edu.cn (W.Z.); 22033100275@stu.hebmu.edu.cn (R.S.); 16000557@hebmu.edu.cn (Y.L.); 24031100109@stu.hebmu.edu.cn (G.Z.); 22033100268@stu.hebmu.edu.cn (X.F.); 23033100293@stu.hebmu.edu.cn (J.C.); 24033100353@stu.hebmu.edu.cn (M.H.); 24033100366@stu.hebmu.edu.cn (Y.A.); 18200713@hebmu.edu.cn (R.M.)

**Keywords:** stress, oligodendrocyte, demyelination, cholesterol, differentiation

## Abstract

Stress-induced demyelination resulting from oligodendrocyte (OLG) dysfunction is one of the key pathological mechanisms of depression, yet its dynamic regulatory network remains unclear. This study integrates single-cell transcriptomics, lineage tracing, and functional interventions to uncover a temporally disordered OLG cholesterol metabolism in a restraint stress mouse model: After 3 days of stress, upregulation of efflux genes Abca1/Abcg1 triggers a compensatory response; however, by day 14, persistent suppression of transport genes (*Apoe*, *Apod*) and homeostatic regulatory genes (*Dhcr24*, *Srebf2*, etc.) leads to intracellular accumulation of “ineffective cholesterol”, with compensatory activation of the AMPK pathway unable to restore cholesterol conversion into myelin. Pseudotime analysis further reveals that stress alters OLG differentiation trajectories, decreasing the proportion of mature OLGs and causing immature precursors to abnormally stall at the late pre-differentiation stage, resulting in myelin regeneration failure. Moreover, an immune OLG_C10 subpopulation expressing complement component *C3* and *P2ry12* is identified, indicating that OLGs may contribute to neuroinflammatory cascades through immune reprogramming. In summary, these findings reveal a novel mechanism from the dynamic perspective of OLGs, in which the interplay of “metabolic imbalance, differentiation blockade, and immune activation” collaboratively drives stress-induced demyelination, providing a theoretical foundation for depression treatment targeting OLG functional restoration.

## 1. Introduction

Depression, as one of the leading causes of disability worldwide, has long been studied with a focus on abnormalities in neuronal synaptic plasticity and imbalances in monoamine neurotransmitters [1,2]. However, recent neuroimaging and postmortem studies have revealed widespread reductions in white matter integrity and myelin structural damage in patients with depression [3,4,5], suggesting that oligodendrocyte (OLG)-mediated myelin homeostasis disruption may play a critical role in disease pathogenesis. As the central nervous system’s primary myelin-forming cells, OLGs not only insulate axons but also influence neural circuit plasticity through noncanonical roles such as metabolic coupling and immune regulation [6,7]. Notably, stress (a core trigger of depression) can significantly reduce OLG density in emotion-related brain regions such as the prefrontal cortex and hippocampus in rodents [8], yet the molecular mechanisms underlying this phenomenon and its causal relationship with depressive behaviors remain largely unknown.

Recent studies on stress-induced depression have predominantly examined the direct neuronal damage caused by stress, neglecting the heightened sensitivity of OLGs, as “metabolic-immune interface cells”, to microenvironmental disturbances [6,9,10,11]. Clinical evidence indicates that cholesterol transport genes (*ApoE*) are abnormally expressed in the brains of depression patients, and stress induces cholesterol accumulation in OLGs in animal models [12,13,14]; however, the spatiotemporal characteristics of this phenomenon and its direct causal link to myelin regeneration failure remain unresolved. Furthermore, OLG differentiation impairment is recognized as a crucial mechanism in demyelinating diseases [15,16], but whether stress disrupts regeneration by reshaping differentiation trajectories rather than simply suppressing differentiation remains unexplored at the single-cell level. Crucially, OLGs are highly heterogeneous, and it remains unknown whether stress selectively activates subpopulations with immune-regulatory or metabolic specialization. Additionally, the hypothalamus, as a central brain region coordinating autonomic, endocrine, and behavioral responses to stress, plays a crucial role in maintaining homeostasis. However, little is known about the changes in OLG within the hypothalamus induced by stress.

In response to these issues, this study utilizes a mouse model of acute and chronic restraint stress—an established model for replicating psychological and physical stress [17], which our research has confirmed to induce typical depression-like behaviors [18]. Moving beyond the traditional neuron-centric perspective, we integrate single-nucleus RNA sequencing (snRNA-seq), lineage tracing, and functional interventions, focusing on OLGs to reveal that cholesterol homeostasis imbalance is a key driver of stress-induced demyelination. Furthermore, it elucidates the spatiotemporal characteristics of OLG differentiation trajectory shifts, providing a dynamic cellular map for understanding myelin regeneration impairment. Finally, this study uncovers the significant involvement of immune OLG subpopulations in stress responses, paving the way for research into the immuno-metabolic mechanisms of depression and establishing a theoretical basis for OLG-targeted antidepressant strategies.

## 2. Results

### 2.1. Stress-Induced OLG Cholesterol Dysregulation Is Associated with Demyelination

**Figure 1:**
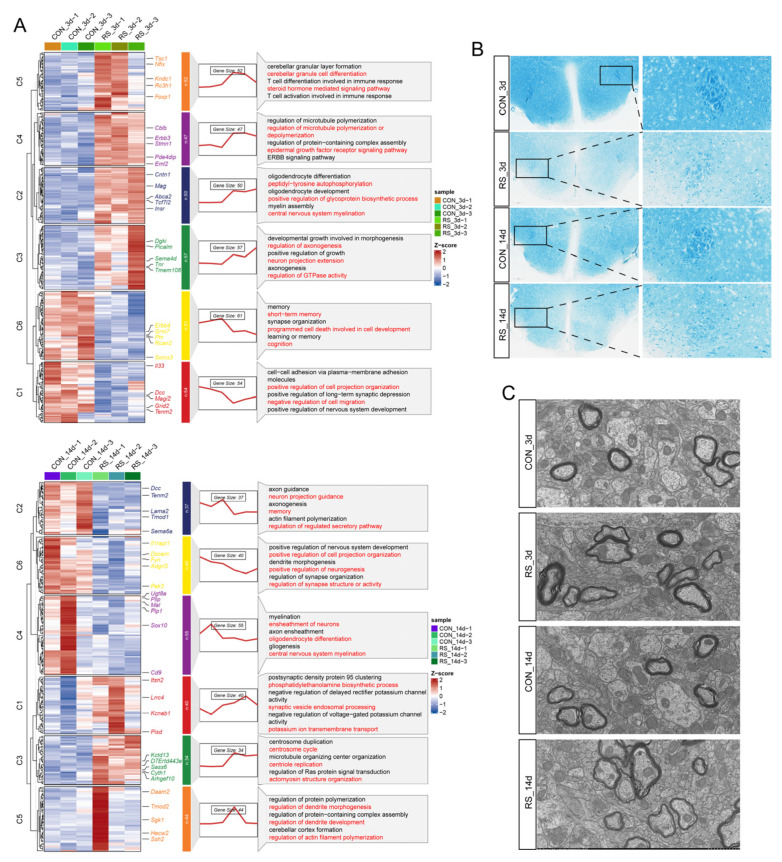
Stress disrupts OLGs’ function, causing demyelination. (**A**) Heatmap of DEGs in OLG under different stress durations, showing expression trends and functional annotations. (**B**) LFB myelin staining in the hypothalamus. Stressed mice showed decreased blue staining and reduced myelin density, especially at 14 days of stress. Left bar = 200 µm, right bar = 50 µm. (**C**) TEM images of myelin. Some myelin showed folding or unraveling and reduced thickness, consistent with demyelination. Bar = 1 µm.

**Figure 2:**
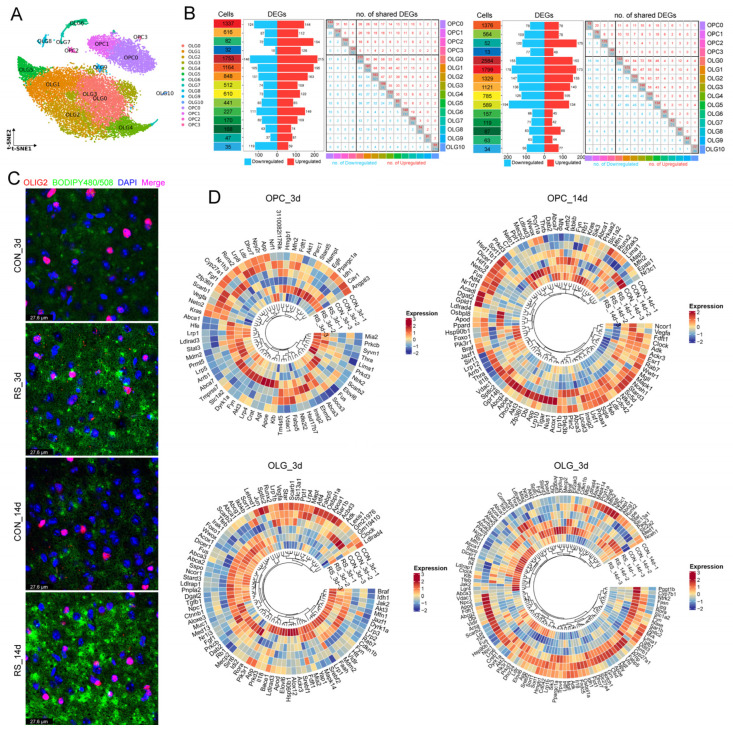
Subdivision of OLGs and abnormal cholesterol metabolism. (**A**) t-SNE plots of re-clustered OPCs and OLGs. OPCs were re-clustered into four subpopulations, and OLGs into 11. (**B**) Overview of DEGs in OLG subpopulations after 3 and 14 days of stress, showing cell counts, up/downregulated DEGs, and shared changes across subpopulations. (**C**) Co-IF staining of OLGs (OLIG2+) with cholesterol (BODIPY 480/508), showing cholesterol accumulation around OLG nuclei, particularly after 14 days of stress. *n* = 6, Bar = 27.6 µm. (**D**) Heatmap of cholesterol-related gene expression in OPCs and OLGs under different stress durations (data scaled by z-score; *n* = 3). The cholesterol genes are sourced from the NCBI database.

**Figure 3:**
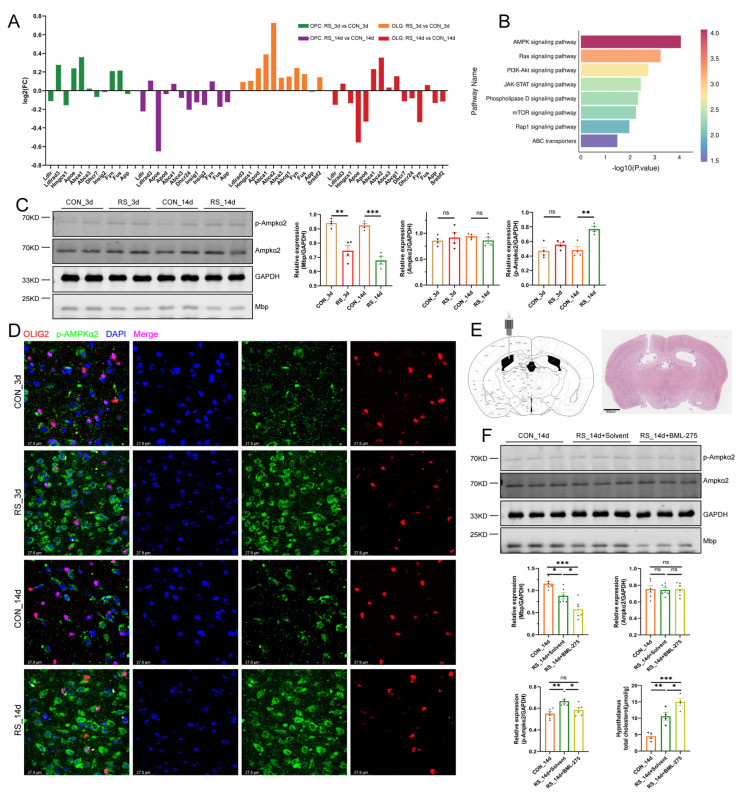
AMPK pathway mediates abnormal cholesterol metabolism in OLGs. (**A**) Fold change in cholesterol metabolism genes in OPCs and OLGs under stress. Y-axis shows gene log2fc values. Cholesterol genes in cell were selected based on a *p* < 0.05. (**B**) KEGG pathway enrichment for upregulated DEGs in OLGs after 14 days of stress, focusing on environmental-information-processing pathways. (**C**) Western blot images and densitometric quantification of Ampkα2, *p*-Ampkα2, and Mbp in the hypothalamus. *n* = 4, values are mean ± SEM, ** *p* < 0.01, *** *p* < 0.001 vs. control. (**D**) Co-IF staining of OLGs (OLIG2+) and p-Ampkα2 in the hypothalamus (*n* = 6). Stress increases p-Ampkα2 expression around OLG nuclei at different time points, Bar = 27.6 µm. (**E**) Microinjection schematic into the lateral ventricle of mice (top: injection diagram, bottom: hematoxylin and eosin (HE) staining). Bar = 800 µm. (**F**) Western blot images and quantification of Ampkα2, p-Ampkα2, and Mbp levels (*n* = 6) in the hypothalamus, with cholesterol levels (*n* = 4) measured. The RS_14d+BML-275 group showed lower p-Ampkα2 and Mbp levels, and increased cholesterol compared to the control. * *p* < 0.05, ** *p* < 0.01, *** *p* < 0.001.

To investigate the effects of stress at different time points on hypothalamic OLGs, we performed snRNA-seq on hypothalamic tissues from 12 mice (3 per group). After quality control and quantification, a total of 18,713 OLG nuclei were obtained, which were further classified into OLGs (14,623) and OLG precursor cells (4090) based on their differentiation trajectories. Subsequently, we employed the Bimod algorithm and ClusterGVis to analyze the impact of stress on the transcriptomes of OPC and OLG. After 3 days of stress, we identified 279 differentially expressed genes (DEGs) in OPC and 321 DEGs in OLG; after 14 days, 150 DEGs were identified in OPC and 250 in OLG (Figure 1A; Supplementary Figure S1A). These DEGs were categorized into six clusters. The processes associated with OPC included synaptic assembly, synaptic organization, and unit assembly components, while the processes related to OLG encompassed myelin assembly, myelination, OLG differentiation, and central nervous system myelination (Figure 1A; Supplementary Figure S1A). This finding is consistent with previous reports of demyelination and synaptic defects in stressed mice and patients with major depressive disorder [4,19,20]. Luxol fast blue (LFB) myelin staining and transmission electron microscope (TEM) further demonstrated that stress leads to marked demyelination in mice, especially after 14 days (Figure 1B,C). We re-clustered OPC and OLG, identifying 15 subpopulations (4 for OPC and 11 for OLG). Notably, OPC_C2, OLG_C0, OLG_C1, OLG_C2, and OLG_C5 exhibited a significant number of DEGs after stress, which were primarily enriched in myelin, synaptic function, and cholesterol metabolism (Figure 2A,B; Supplementary Figure S1B). Additionally, cholesterol immunofluorescence (IF) staining in OLG showed that stress increases intracellular cholesterol levels (Figure 2C), consistent with the overall increase in cholesterol content throughout the hypothalamus after stress. Therefore, we hypothesize that stress-induced cholesterol metabolic reprogramming may significantly contribute to demyelination. Accordingly, we examined the cholesterol-regulating genes in OPC and OLG, discovering notable cellular heterogeneity and variations in their responses to stress over time (Figure 2D). Specifically, stress influences cholesterol synthesis (*Ldlr*, *Hmgcs1*), and enhances cholesterol efflux (*Abca1*, *Abca2*, *Abca3*, *Abcg1*). In contrast, transport genes (*Apoe*, *Apod*) show temporal differences in their responses (increased at 3 days and decreased at 14 days). Moreover, stress reduces the expression of cholesterol homeostasis genes (*Dhcr7*, *Dhcr24*, *Fyn*, *App*, *Srebf2*) (Figure 3A). These data indicate that stress disrupts cholesterol metabolism and leads to cholesterol accumulation.

### 2.2. AMPK Alleviates Stress-Induced OLG Cholesterol Dysregulation and Demyelination

**Figure 4:**
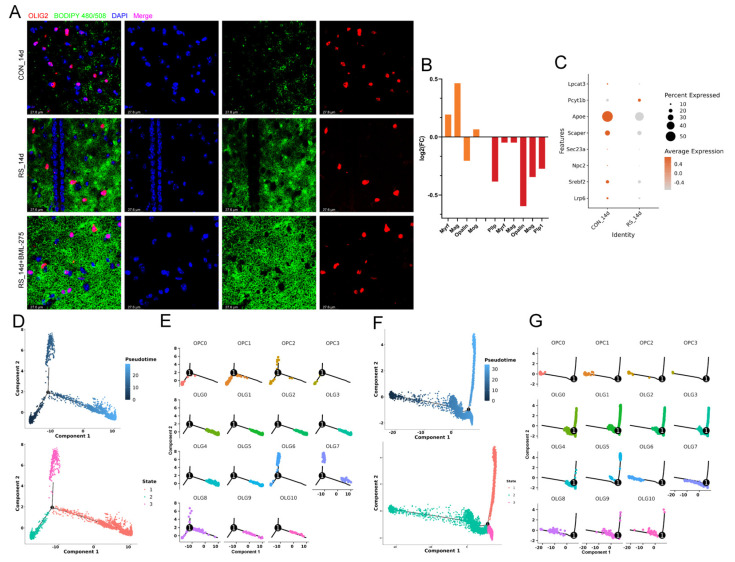
Stress induced abnormal cholesterol transport and differentiation trajectory in OLGs. (**A**) Co-IF staining of OLGs (OLIG2+) and cholesterol (BODIPY 480/508) in the hypothalamus (*n* = 6). The RS_14d+BML-275 group shows increased cholesterol accumulation around OLG nuclei, Bar = 27.6 µm. (**B**) Differential fold changes in myelin-related genes in OLGs at different stress timepoints. Selection for myelin genes in cell is based on DEGs with *p* < 0.05. (**C**) Dot plot of cholesterol transport gene expression to the ER in OLGs after 14 days of stress, showing reduced expression of cholesterol transport genes (*Lrp6*, *Srebf2*, *Npc2*, *Sec23a*, *Scaper*, *Apoe*, *Lpcat3*). (**D**) Differentiation trajectory of OLGs in the 3-day stress group (top) and cell monocle_state tree structure trajectory (bottom). The differentiation trajectory arranges cells based on their distance from the default starting point of the pseudotime axis using Monocle2. (**E**) Tree-structured trajectory distribution of OLG subpopulations in the 3-day stress group. (**F**) Differentiation trajectory of OLGs in the 14-day stress group (top) and the cell monocle_state tree structure trajectory (bottom). (**G**) OLG subpopulation tree structure trajectory in the 14-day stress group.

Studies indicate that activation of the AMPK signaling pathway reduces cholesterol production and enhances cholesterol efflux, thereby mitigating accumulation [21,22]. Our analysis revealed significant activation of the AMPK pathway in OLG from mice after 14 days of stress (*p*-value = 8.74 × 10^−5^) (Figure 3B), with increased expression of related genes (*Adipor2*, *Cab39l*, *Scd3*). Western blot and IF further confirmed the activation of the AMPK signaling pathway during demyelination induced by stress (Figure 3C,D). Administration of BML-275 into the lateral ventricle to inhibit the AMPK signaling pathway (Figure 3E) led to greater cholesterol accumulation in the hypothalamus and OLG, as well as more pronounced demyelination (Figure 3F and Figure 4A). These findings indicate that although stress may alleviate cholesterol accumulation to some extent through AMPK activation, it cannot prevent the abnormal cholesterol buildup that triggers demyelination. Furthermore, cholesterol is a crucial structural component of myelin, necessary for its growth and the wrapping of axons. Ensuring sufficient cholesterol levels in OLG is critical for myelin formation and regeneration following myelin damage [23]. Analyzing genes associated with myelin formation (*Pllp*, *Myrf*, *Mag*, *Opalin*, *Mog*, *Plp1*) showed that stress after 14 days reduces the expression of these genes (Figure 4B). This indicates that the conversion of cholesterol into myelin is hindered, implying that the increased intracellular cholesterol is not adequately transformed into functional myelin to replenish damaged myelin. Upon investigation, we found that the expression of genes (*Lrp6*, *Srebf2*, *Npc2*, *Sec23a*, *Scaper*, *Apoe*, *Lpcat3*) responsible for cholesterol transport to the endoplasmic reticulum (ER) in OLG is decreased (Figure 4C). This suggests that the processing of cholesterol into myelin in the ER is impaired.

### 2.3. Stress-Induced Impairment of OLG and OPC Differentiation Exacerbates Demyelination

**Figure 5:**
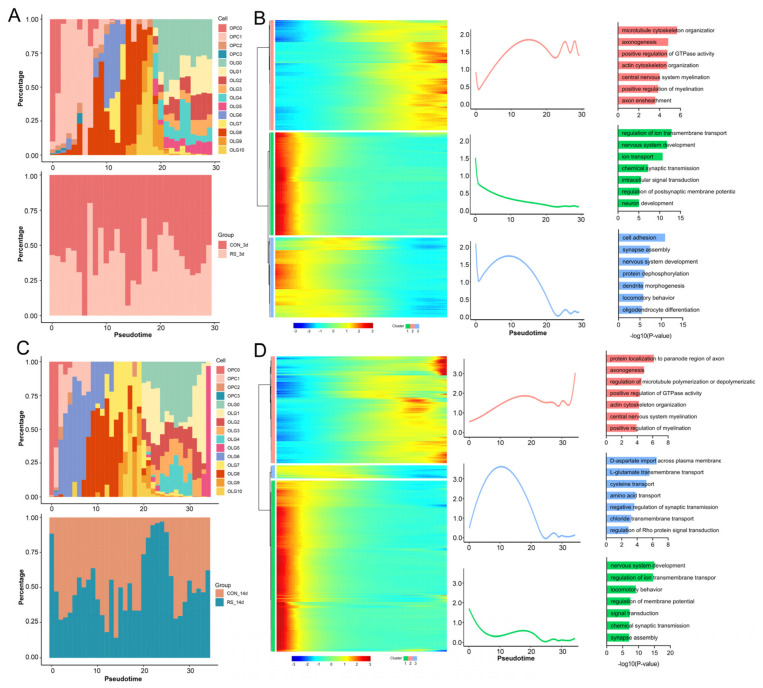
Stress causes a deviation in the differentiation trajectory of OLGs. (**A**) Pseudotime analysis of OLG subpopulations in stress, ordered on the pseudotime axis. Bars are shown with different sub-cell types (top) and groups (bottom). (**B**) Heatmaps showing gene expression profiles along pseudotime for different OLG subpopulations and 3-day stress groups, divided into three clusters with associated GO terms. (**C**) Pseudotime analysis of OLG subpopulations along the pseudotime axis. (**D**) Heatmaps of gene expression profiles across pseudotime in OLG subpopulations (CON_14d and RS_14d), with enriched GO terms listed.

In chronic demyelinating diseases, promoting the proliferation and differentiation of OPCs is beneficial for the regeneration of damaged myelin [23]. Thus, we examined the lineage trajectories of the 15 subpopulations of OPC and OLG. The differentiation pathways observed at 3 and 14 days of stress were largely similar, comprising one node and three states, with each subpopulation appearing at different points in the differentiation process. Notably, OPC_C0–OPC_C3 are at the initiation of differentiation, while OLG_C0–OLG_C3 are at the endpoint, indicating mature OLGs involved in myelin formation. OLG_C4–OLG_C10 represent the transition from precursor to mature OLGs (Figure 4D–G), consistent with previously documented OLGs’ differentiation processes [24,25]. A dynamic comparison of cell composition shows that the pseudotime cell composition is similar between the stress and control groups, but the proportion of OPCs, directed/newly formed OLGs, and myelinating/mature OLGs decreases after stress (Figure 5A,C). Additionally, the proportion of precursor cells for myelinating/mature OLGs after stress is higher than in the control group (around 20 in pseudotime at 3 days; 20–25 at 14 days), especially at 14 days post-stress (Figure 5A,C). This suggests that stress-induced OLG activation and differentiation preferentially convert to precursor cells for myelinating/mature OLGs rather than functional OLGs. To better understand how stress influences OLG differentiation, we identified three distinct gene clusters based on the top 1000 cell-type-specific genes and pseudotime differentiation waves, mapping the continuous wave of gene expression (wave-DEGs) (Figure 5B,D). At 3 days post-stress, Cluster 1 of wave-DEGs was expressed in OPC_C0–OPC_C3 and gradually downregulated along the trajectory, primarily involving biological processes related to the regulation of ion transmembrane transport and nervous system development. Cluster 2 of wave-DEGs gradually upregulated along the differentiation trajectory, primarily expressed in OLG_C0–OLG_C3, with biological processes mainly involving microtubule cytoskeleton organization, axonogenesis, and central nervous system myelination. Cluster 3 wave-DEGs showed high expression initially, then briefly declined before increasing again, particularly in directed/newly formed OLGs responding to differentiation stimuli, and subsequently dropping sharply. This suggests their expression aligns with re-entry into differentiation stages, primarily involved in processes like nervous system development, dendrite morphogenesis, and OLG differentiation (Figure 5B). At 14 days after stress, the cell-type-specific gene expression profile along the differentiation trajectory was generally similar to that at 3 days, with notable differences in Cluster 3 wave-DEGs. This cluster showed lower initial expression, which increased over time and then sharply decreased after cellular differentiation. High-expression cell types were also directed/newly formed OLGs, but their biological processes primarily involved D-aspartate import across the plasma membrane and L-glutamate transmembrane transport (Figure 5D). In summary, these findings suggest that stress results in a decrease in OPCs and OLGs, while the body attempts to reactivate OLG differentiation continuity. However, this process predominantly leads to the formation of precursor cells for myelinating/mature OLGs, rather than fully functional OLGs, preventing effective repair of damaged myelin.

### 2.4. Identification of Immune Subpopulations of OLGs

**Figure 6:**
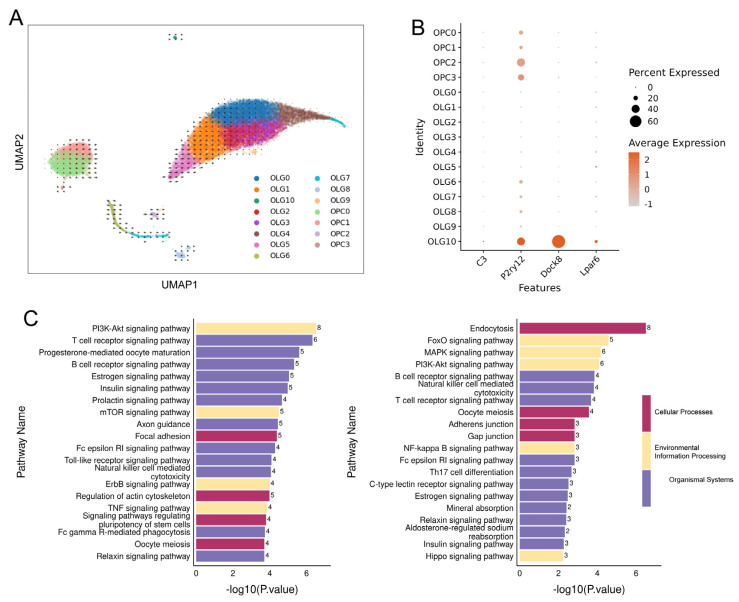
Identification of immune OLGs subpopulations. (**A**) UMAP dimensionality reduction clustering results of OLGs. The cells are colored according to OLG subpopulations, and arrows indicate the direction of individual cell movement and RNA velocity. (**B**) Distribution of immune-related genes in OLG subpopulations. (**C**) KEGG pathway enrichment of DEGs in OLG_C10 under different stress conditions. Left: 3-day stress group; right: 14-day stress group.

Furthermore, we were astonished to discover that OLG_C10 closely aligns with the “immune OLGs” defined by Jäkel et al. [26], as this subpopulation is significantly separated from others and specifically expresses immune-related genes like *C3*, *P2ry12*, *Dock8*, and *Lpar6* (Figure 6A,B). This finding aligns with the results observed by Nagy et al. in their snRNA study of the prefrontal cortex in patients with major depressive disorder [5]. Compared with the control group, the DEGs of OLG_C10 were primarily enriched in the PI3K-Akt signaling pathway, T cell receptor signaling pathway, and B cell receptor signaling pathway under 3-day stress; at 14 days of stress, DEGs were mainly enriched in Endocytosis, the FoxO signaling pathway, the PI3K-Akt signaling pathway, and the B cell receptor signaling pathway (Figure 6C).

## 3. Discussion

By integrating transcriptomic analysis, cell lineage tracing, and functional validation experiments, this study systematically reveals the molecular mechanism through which stress-induced OLG cholesterol metabolism dysregulation and differentiation impairment jointly drive demyelination. The findings not only expand the pathological understanding of stress-induced demyelination but also provide potential intervention targets for myelin repair in depression and other psychiatric disorders.

Although the association between cholesterol accumulation and demyelination has been reported [27,28], this study is the first to reveal, through single-cell dynamic analysis, the temporal disruption of the OLG cholesterol metabolic network by stress. During early-stage stress (3 days), OLGs attempt to maintain homeostasis by upregulating cholesterol efflux genes (e.g., *Abca1*, *Abcg1*), suggesting the activation of a compensatory protective mechanism. However, under chronic stress (14 days), the sustained suppression of cholesterol transport genes (*Apoe*, *Apod*, etc.) and homeostasis-regulating genes (*Dhcr24*, *Srebf2*, etc.) indicates that the compensatory capacity of OLGs becomes “exhausted” over prolonged stress, ultimately leading to abnormal cholesterol accumulation in the cytoplasm. This phenomenon may reflect stress-induced disruption of cellular metabolic resilience, where early adaptive responses gradually fail during the chronic phase, leading to an irreversible pathological state. Notably, although the AMPK pathway is activated in the late phase of stress and is theoretically capable of alleviating cholesterol accumulation by inhibiting cholesterol synthesis and promoting efflux [21,29], experimental inhibition of AMPK paradoxically exacerbates demyelination, suggesting that its compensatory function under stress is limited. This may be related to the spatiotemporal specificity of AMPK activation: early stress-induced AMPK activation may exert a protective effect by clearing excess cholesterol, whereas prolonged activation may interfere with cholesterol conversion into myelin. Combined with the downregulation of endoplasmic reticulum cholesterol transport genes (*Lrp6*, *Scaper*, etc.) in OLGs, we propose that stress-induced metabolic dysregulation results in abnormal cytoplasmic cholesterol accumulation, while impaired endoplasmic reticulum processing capacity further hinders its conversion into functional myelin lipids, forming an “ineffective cholesterol pool” that ultimately leads to myelin regeneration failure.

Utilizing pseudotime analysis, this study elucidates for the first time the cellular dynamics through which stress exacerbates demyelination by remodeling OLG differentiation trajectories. Although stress does not alter the overall differentiation structure of the OLG lineage, it significantly reduces the proportion of mature OLGs while leading to an accumulation of differentiation precursors, indicating that stress induces OLG differentiation arrest at an immature functional state. The molecular mechanisms contributing to this phenomenon may include the following: (1) functional deficiencies in immature OLGs that compromise myelin synthesis efficiency [16]; (2) abnormal accumulation of differentiation precursors, potentially generating negative feedback via the secretion of inhibitory factors (e.g., TGF-β), thereby further hindering myelin regeneration [30,31]. Subsequent dynamic analysis of wave-DEGs provides a molecular explanation for this phenomenon. At 3 days of stress, the gene expression patterns regulating ion transport (Cluster 1) and axon formation (Cluster 2) still maintain the basic differentiation process. However, by 14 days, changes in the expression pattern of Cluster 3 genes (involved in glutamate transport) may interfere with the metabolic coupling between OLGs and neurons (e.g., the glutamate–glutamine cycle), leading to insufficient energy supply for OLGs and thus blocking terminal differentiation [24,32,33]. This finding parallels the pathological characteristic of reduced synaptic plasticity observed in depression patients [34,35], suggesting that OLG differentiation impairment may indirectly affect neuronal function through the “metabolism-synapse” axis, forming a vicious cycle. These results imply that stress inflicts dual damage on OLGs by not only diminishing their quantity but also compromising the functional efficiency of the remaining population through differentiation trajectory alterations.

Finally, this study identifies that the proportion of the OLG_C10 subpopulation increases at 3 days of stress and is enriched in immune-related pathways (e.g., PI3K-Akt, T/B cell receptor signaling), suggesting that stress may participate in neuroinflammatory responses by activating the immunomodulatory functions of OLGs. This finding expands the role of OLGs from the traditional “myelin-supporting cells” to “immune regulators”, suggesting that stress may reshape the immune properties of OLGs, working in concert with microglia and astrocytes to sculpt the neuroinflammatory microenvironment [5,36]. This correlates with the elevated neuroinflammatory markers observed in the brains of depression patients [5]. However, at 14 days of stress, the proportion of this subpopulation decreases, and the FoxO pathway is downregulated, potentially indicating a shift from a pro-inflammatory to a pro-survival phenotype. However, whether this functional transition contributes to compensatory repair remains to be further validated.

In summary, this study elucidates the multidimensional regulatory network of “metabolism-differentiation-immunity” in stress-induced demyelination, laying a theoretical foundation for developing precise intervention strategies targeting OLG functional restoration. However, this study is currently limited by the use of animal models, which cannot fully replicate the complex social environment of chronic stress in humans, and it has not yet clarified the causal relationship between cholesterol accumulation and differentiation impairment. Future studies should integrate spatial transcriptomics and live imaging techniques to dynamically resolve the spatiotemporal coupling mechanisms of cholesterol metabolism and differentiation processes.

## 4. Materials and Methods

### 4.1. Establishment of the Stress Experimental Model

Male C57BL/6N mice (7–8 weeks old, 22 ± 2 g) were obtained from Beijing Vital River Laboratory Animal Technology Co., Ltd. (Beijing, China), and acclimated for 7 days under SPF-grade barrier conditions (temperature: 22 ± 2 °C, 12 h/12 h light/dark cycle). All animal procedures were approved by the Animal Ethics and Welfare Committee of Hebei Medical University (Approval No. IACUC-Hebmu-2023011). The mice were randomly assigned to four groups: CON_3d (control, 3 days), CON_14d (control, 14 days), RS_3d (restraint stress, 3 days), and RS_14d (restraint stress, 14 days). The stress groups underwent 6 h of daily restraint stress (the mice were confined in a cylindrical plastic tube (3 cm in diameter, 10 cm in length) with ventilation holes), whereas the control groups experienced only food and water deprivation for the corresponding period. At the endpoint, the mice were anesthetized via intraperitoneal injection of 2% pentobarbital sodium (3.2 mL/kg), followed by retro-orbital blood collection and euthanasia via cervical dislocation. The whole brain was fixed in 4% paraformaldehyde, or the hypothalamus was rapidly frozen in liquid nitrogen and stored at −80 °C for subsequent experiments.

Additionally, C57BL/6N mice were randomly divided into three groups: control, stress + vehicle (normal saline containing 0.01% DMSO), and stress + AMPK inhibitor. The AMPK inhibitor BML-275 (dorsomorphin dihydrochloride, Cat# HY-13418, MedChemExpress, Monmouth Junction, NJ, USA) was delivered via intracerebroventricular stereotaxic injection (due to the large dosage, direct injection into hypothalamic tissue will excessively damage normal tissue and affect its function). Following anesthesia and fixation, stereotaxic injection was performed based on the mouse brain atlas (coordinates: AP: −0.820 mm; ML: −1.486 mm; DV: −1.969 mm) using a microinjection cannula (Shanghai Yuyan Instruments Co., Ltd., Shanghai, China, Model: L1 = 2 mm, L3/L4 = 0 mm). BML-275 solution was administered at a dose of 10 mg/kg (5 μL per injection) one hour before daily restraint. The restraint stress model was established as described above.

### 4.2. HE Staining

Mouse brain tissues were fixed in 4% paraformaldehyde for 24 h, then embedded in paraffin, and coronal sections from 4.54 mm to 0.88 mm (relative to the interaural line) were continuously cut at 4 μm thickness. The sections were deparaffinized in xylene, rehydrated through a graded ethanol series, stained with hematoxylin for 5 s, rinsed in running water for 10 min, differentiated with 0.5% hydrochloric acid ethanol for 3 s, and counterstained with eosin for 3 s after bluing. The sections were dehydrated through a graded ethanol series, cleared in xylene, mounted with neutral resin, and examined under a light microscope.

### 4.3. LFB Myelin Staining

Paraffin sections were deparaffinized in a xylene gradient, rehydrated through a graded ethanol series, and equilibrated in 95% ethanol. The sections were stained with LFB solution at room temperature for 20 h, followed by differentiation in 95% ethanol to remove nonspecific staining. The sections were then differentiated in 0.05% lithium carbonate 10% ethanol for 15 s, followed by 70% ethanol for 30 s until a clear contrast between gray and white matter was achieved. After dehydration through a graded ethanol series and clearing with xylene, the sections were mounted with neutral resin.

### 4.4. IF Staining

Paraffin sections were deparaffinized with xylene and rehydrated through a graded ethanol series, followed by antigen retrieval using microwave-mediated citrate buffer (10 mM, pH 6.0). Endogenous peroxidase activity was blocked with 3% H_2_O_2_ for 30 min at room temperature. After three washes with PBS, the sections were blocked with 10% normal goat serum at 37 °C for 45 min. The sections were incubated overnight at 4 °C with a primary antibody against (Olig2 (1:500) and p-AMPKα2 (1:200) antibodies were obtained from Huaan Biotechnology (Hangzhou, Zhejiang, China), while Bodipy480/508 (1:1000) was purchased from Cayman Chemical (Ann Arbor, MI, USA)). After PBS washes, the sections were incubated for 1 h at 37 °C in the dark with species-specific secondary antibodies (DyLight™ 594-labeled anti-rabbit IgG or DyLight™ 488-labeled anti-mouse IgG, 1:500 dilution). After thorough PBS washes, the sections were mounted using FluoroMount-G^®^ with DAPI (Homewood, AL, USA).

### 4.5. TEM

Ultrathin sections of hypothalamic tissue were prepared for TEM to examine ultrastructural changes. Tissue samples were fixed in 2.5% glutaraldehyde at 4 °C overnight, followed by post-fixation in 1% osmium tetroxide for 2 h at room temperature. After graded ethanol dehydration, the samples were embedded in epoxy resin and polymerized at 60 °C for 48 h. Ultrathin sections (70 nm) were cut using an ultramicrotome, collected on copper grids, and stained with uranyl acetate and lead citrate. The sections were then examined under a transmission electron microscope at 80 kV, and images were acquired for further analysis.

### 4.6. Western Blotting

Mouse hypothalamic tissue was lysed on ice in RIPA lysis buffer containing protease and phosphatase inhibitors, then centrifuged at 4 °C to collect the supernatant. Protein concentration was measured using the BCA assay. Equal protein samples were separated by SDS-PAGE and transferred onto PVDF membranes using a semi-dry transfer system (Trans-Blot Turbo). The membranes were blocked with 5% skim milk for 1 h, then incubated with primary antibodies (overnight at 4 °C; Mbp (1:500) was purchased from Santa Cruz Biotechnology, Inc. (Dallas, TX, USA), while Ampkα2 (1:1000) and p-AMPKα2 (1:500) were obtained from Huaan Biotechnology (Hangzhou, Zhejiang, China) followed by fluorescently labeled secondary antibodies (DyLight 680 anti-mouse or IRDye 680RD anti-rabbit, 1 h at 37 °C). After extensive TBST washing, the LI-COR Odyssey dual-color infrared imaging system (Lincoln, NE, USA) was used for detection. The relative expression levels of target proteins were quantified using ImageJ software (v1.8.0, NIH).

### 4.7. Hypothalamic snRNA-seq

Hypothalamus tissue was collected from healthy and stressed male C57BL/6N mice. The tissue was transferred to an ice-cold, RNase-free culture dish containing calcium- and magnesium-free PBS, cut into ~0.5 mm^2^ pieces, and washed to remove blood and fat. For dissociation, the tissues were incubated in a digestion solution (0.35% collagenase IV, 2 mg/mL papain, 120 U/mL DNase I) at 37 °C with shaking (100 rpm) for 20 min. Digestion was halted with PBS containing 10% fetal bovine serum, followed by pipetting 5–10 times. The suspension was filtered through stacked 70–30 μm cell strainers and centrifuged at 300 g for 5 min at 4 °C. The pellet was resuspended in 100 μL PBS (0.04% BSA), treated with red blood cell lysis buffer, and incubated for 2 min at room temperature or on ice. After centrifugation (300× *g*, 5 min, room temperature), the dead cells were removed using Dead Cell Removal MicroBeads (Cologne, Germany) and the Miltenyi^®^ Dead Cell Removal Kit (Munich, Germany). The suspension was then washed twice in PBS (0.04% BSA) and centrifuged at 300× *g* for 3 min at 4 °C. The final cell pellet was resuspended in 50 μL PBS (0.04% BSA). Cell viability (>85%) was assessed via trypan blue exclusion, and single-cell suspensions were counted using a Countess II Automated Cell Counter (Carlsbad, CA, USA), adjusting the concentration to 700–1200 cells/μL.

Single-cell suspensions were loaded onto the 10× Chromium system to capture 5000 single cells following the 10× Genomics Chromium Single-Cell 3’ Kit (V3) protocol. cDNA amplification and library construction were performed according to the standard protocol. Libraries were sequenced on an Illumina NovaSeq 6000 by LC-Bio Technology Co., Ltd., (Hangzhou, China) at a minimum depth of 20,000 reads per cell.

### 4.8. Bioinformatics Analysis

Sequencing data were demultiplexed and converted to FASTQ format using Illumina bcl2fastq (v2.20). The Cell Ranger pipeline (v3.1.0) was used for sample demultiplexing, barcode processing, and single-cell 3’ gene counting. scRNA-seq data were aligned to the Ensembl GRCh38/GRCm38 reference genome. A total of 139,686 single cells from 6 healthy mice (three from CON_3d, three from CON_14d) and 6 stressed mice (three from RS_3d, three from RS_14d) were processed using the 10× Genomics Chromium Single Cell 3’ Solution.

Cell Ranger output was analyzed in Seurat (v3.1.1) for dimensional reduction, clustering, and scRNA-seq data analysis. Quality control thresholds were applied: genes expressed in fewer than three cells were removed, genes per cell were set between 500 and 5000, UMI counts were filtered for <500, and mitochondrial gene expression was limited to <25%. For visualization, dimensionality reduction was performed using UMAP. The steps included the following: (1) normalizing gene expression using Seurat’s LogNormalize method; (2) performing PCA and selecting the top 10 principal components for clustering and UMAP analysis; (3) identifying clusters using the weighted Shared Nearest Neighbor (SNN) graph-based method; (4) detecting marker genes for each cluster via the Wilcoxon rank-sum test (default: “bimod”) using Seurat’s FindAllMarkers function, selecting genes expressed in >10% of cells with an average log fold change >0.26.

### 4.9. Statistical Analysis

All statistical analyses and figure generation were performed using GraphPad Prism 9 (GraphPad Software, Inc., San Diego, CA, USA), with experimental data presented as Mean ± SEM. Based on data distribution, group differences were analyzed using a t-test or one-way ANOVA analysis for normally distributed data, whereas nonparametric tests were applied for non-normally distributed data. Statistical significance was set at *p* < 0.05.

## 5. Conclusions

Through single-cell dynamic analysis, this study demonstrates that stress induces demyelination through the multidimensional interaction of “metabolism-differentiation-immunity” in OLGs. Early stress triggers a compensatory cholesterol efflux response, whereas chronic stress leads to sustained suppression of transport and endoplasmic reticulum processing genes, resulting in the accumulation of “ineffective cholesterol”, which directly impairs functional myelin regeneration. Differentiation trajectory analysis indicates that OLGs are arrested at the precursor-to-mature transition state, unveiling “trajectory deviation” as a novel mechanism of demyelination. Additionally, this study identifies the stress-specifically activated immune OLG_C10 subpopulation, which mediates neuroinflammatory cascades via complement *C3* and *P2ry12*. These findings challenge the traditional neuron-centric paradigm, for the first time constructing a three-dimensional interaction framework of OLG dysfunction, laying a theoretical foundation for synergistic antidepressant strategies targeting cholesterol metabolism remodeling, differentiation promotion, and immune regulation.

## Data Availability

The datasets generated and/or analyzed during the current study are available from the corresponding author on reasonable request.

## Supplementary Materials

The following supporting information can be downloaded at: https://www.mdpi.com/article/10.3390/ijms26083517/s1.
