# Supplementary material for: Stress-Induced Cholesterol Metabolic Dysregulation and Differentiation Trajectory Shift in Oligodendrocytes Synergistically Drive Demyelination
Source: Int J Mol Sci. 2025 Apr 9;26(8):3517. doi: 10.3390/ijms26083517 (PMC12026842; doi:10.3390/ijms26083517)
Supplement: Supplementary file 1 [file ijms-26-03517-s001.zip › ijms-3556412-supplementary.pdf]

Supplemental Information

Stress-Induced Cholesterol Metabolic Dysregulation and Differentiation Trajectory Shift in Oligodendrocytes Synergistically Drive Demyelination

Weihao Zhu[&], Rui Shi[&], Yingmin Li, Guowei Zhang, Xiaowei Feng, Jingze Cong, Mengting He, Yuchuan An, Rufei Ma, Weibo Shi* and Bin Cong*

Hebei Key Laboratory of Forensic Medicine, Collaborative Innovation Center of Forensic Medical Molecular Identification, College of Forensic Medicine, Hebei Medical University, No.361 Zhongshan Dong Road, 050017, Shijiazhuang, China.

[#]Two authors contributed equally to this work.

***Corresponding authors**

Bin Cong, M.D., Ph.D., Tel: 86 311 86266406, E-mail: cong6406@hebmu.edu.cn

Weibo Shi, Ph.D., Tel: 86 311 86261294, E-mail: shiweibo@hebmu.edu.cn

COMPETING INTERESTS

The authors declare no competing interests.

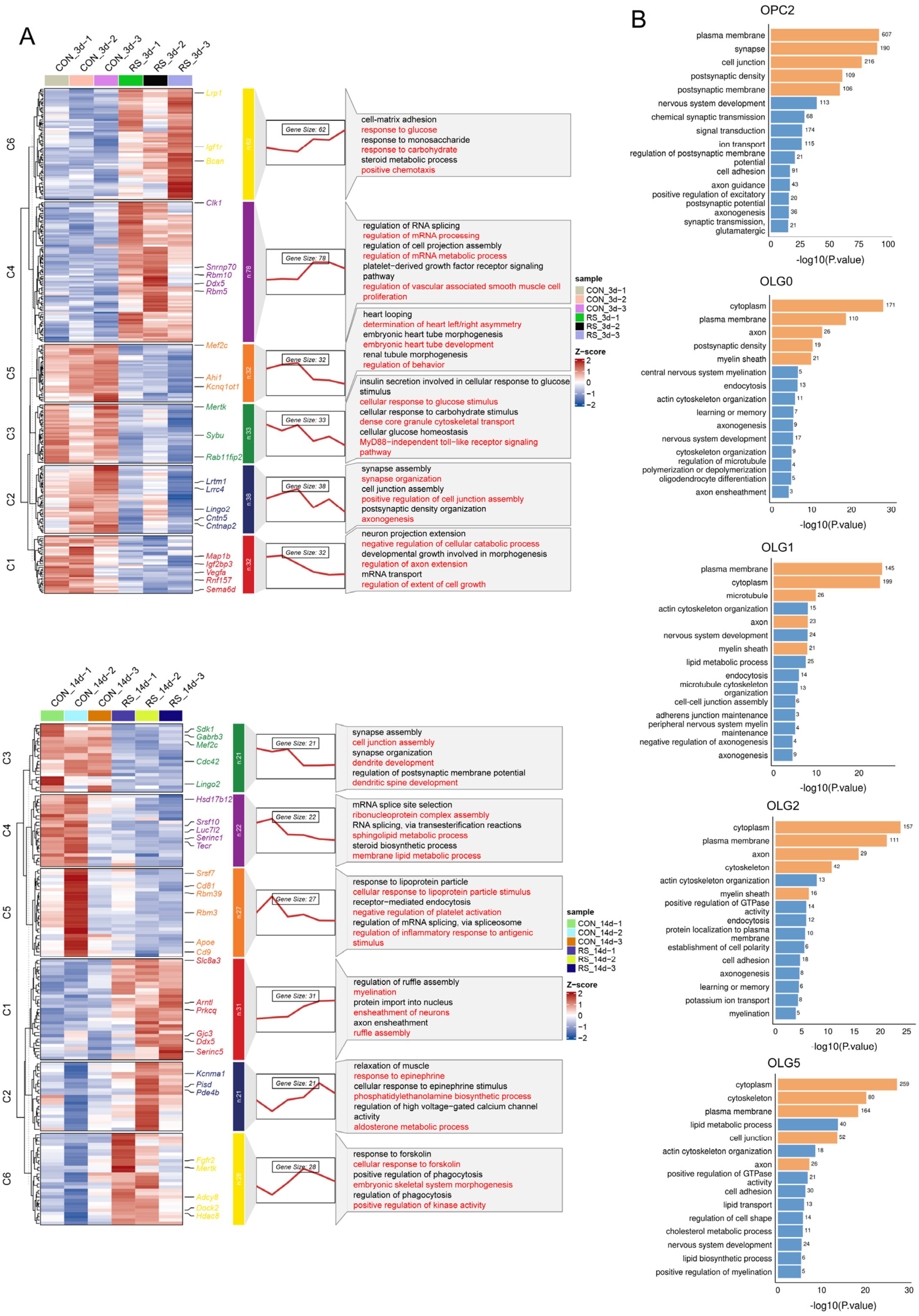

Supplementary Figure S1. Cholesterol abnormalities and demyelination in stress-induced OLGs. (A). Heatmap of DEGs expression, clustering, expression trends, and functional annotation of OPCs under different stress durations. (B). Functional enrichment of DEGs in stress-sensitive OLG subpopulations. DEGs in OLG_C0, OLG_C1, and OLG_C2 are associated with axon, myelin sheath, and CNS myelination. OPC_C2 is linked to synapse, postsynaptic density, and

nervous system development. OLG_C5 is related to axon, lipid metabolism, and cholesterol metabolism.
